# Recellularization of Decellularized Lung Scaffolds Is Enhanced by Dynamic Suspension Culture

**DOI:** 10.1371/journal.pone.0126846

**Published:** 2015-05-11

**Authors:** Aurélie Crabbé, Yulong Liu, Shameema F. Sarker, Nicholas R. Bonenfant, Jennifer Barrila, Zachary D. Borg, James J. Lee, Daniel J. Weiss, Cheryl A. Nickerson

**Affiliations:** 1 The Biodesign Institute, Center for Infectious Diseases and Vaccinology, Arizona State University, Tempe, Arizona, United States of America; 2 Department of Medicine, University of Vermont College of Medicine, Burlington, Vermont, United States of America; 3 Division of Pulmonary Medicine, Department of Biochemistry and Molecular Biology, Mayo Clinic Arizona, Scottsdale, Arizona, United States of America; 4 School of Life Sciences, Arizona State University, Tempe, Arizona, United States of America; Politecnico di Milano, ITALY

## Abstract

Strategies are needed to improve repopulation of decellularized lung scaffolds with stromal and functional epithelial cells. We demonstrate that decellularized mouse lungs recellularized in a dynamic low fluid shear suspension bioreactor, termed the rotating wall vessel (RWV), contained more cells with decreased apoptosis, increased proliferation and enhanced levels of total RNA compared to static recellularization conditions. These results were observed with two relevant mouse cell types: bone marrow-derived mesenchymal stromal (stem) cells (MSCs) and alveolar type II cells (C10). In addition, MSCs cultured in decellularized lungs under static but not bioreactor conditions formed multilayered aggregates. Gene expression and immunohistochemical analyses suggested differentiation of MSCs into collagen I-producing fibroblast-like cells in the bioreactor, indicating enhanced potential for remodeling of the decellularized scaffold matrix. In conclusion, dynamic suspension culture is promising for enhancing repopulation of decellularized lungs, and could contribute to remodeling the extracellular matrix of the scaffolds with subsequent effects on differentiation and functionality of inoculated cells.

## Introduction

Chronic obstructive pulmonary disease (COPD) affects over 64 million people worldwide and is predicted by the World Health Organization to become the third leading cause of mortality by 2030. While allogeneic lung transplantation is the only definitive treatment for the growing number of patients with end-stage lung disease, only one out of four patients on the organ waiting list undergoes transplantation, given the limited availability of donor organs. Moreover, the clinical success of lung transplantation is hampered by lifelong immunosuppression and chronic rejection, reflected in a 10–20% survival rate 10 years post-transplantation [1].

A promising option to increase the donor organ pool is to use allogeneic or xenogeneic decellularized lungs as a scaffold to engineer functional lung tissue *ex vivo* [2–7]. Decellularization of mouse, rat, goat, sheep, pig, non-human primate and human lung tissue has been accomplished with several detergent-based approaches as well as with freeze-thaw cycles, and resulted in three-dimensional (3-D) acellular scaffolds that are generally devoid of detectable residual DNA and nuclei [2–17]. While the decellularized lung scaffolds produced by different methods generally retain major extracellular matrix (ECM) proteins, several ECMs can be less abundant (e.g., collagen I, collagen IV), fragmented (fibronectin) or largely absent (elastin) as compared to the native tissue [2,5,8]. Repopulation of decellularized lungs has been reported using a number of different cell types, including transformed cell lines such as A549 lung adenocarcinoma cells, fetal lung cells, endothelial cells, embryonic stem cells (ESC), fibroblasts, induced pluripotent stem cells (iPSC), primary or immortalized airway and alveolar epithelial cells, and bone marrow or adipose-derived mesenchymal stem cells (MSCs) [2–17]. However, apart from use of fetal lung homogenates in combination with A549 and vascular endothelial cells, only partial recellularization of alveoli, airways and pulmonary vasculature has been achieved [2,8,15,18].

One potential approach to improve recellularization of decellularized lung scaffolds is to use the dynamic rotating wall vessel (RWV) bioreactor, which has been shown to promote growth and differentiation of stem and/or epithelial cells (in the presence or absence of a growth substrate) [19–21]. The RWV is an optimized form of continuous suspension culture wherein cells are cultured in horizontally rotating bioreactors that are completely filled with media [22]. The bioreactor rotation offsets sedimentation, creating a constant, gentle fall of cells and their growth substrate/scaffolds through the culture medium under conditions of physiological fluid shear [23–25], such as those encountered in the interstitium [26]. Under these conditions, gentle media mixing and excellent mass diffusion are obtained [22]. The RWV technology has been used to generate differentiated lung tissue culture models, with important applications in the fields of infectious disease and regenerative medicine [7,22,27,28–31]. Specifically, tumorigenic A549 lung epithelial cells cultured alone and in combination with functional macrophages on the surface of extracellular matrix-coated porous microcarrier beads exhibited phenotypic reversion towards a more normal differentiated phenotype [27,28,31]. Moreover; when challenged with respiratory pathogens and their toxins, these 3-D lung models exhibited a more *in vivo*-like response as compared to conventional 2-D monolayers of the same cell line [27,28]. In the field of lung regenerative medicine, the RWV has been used to differentiate mESCs into multiple distal respiratory epithelial cell types using conditioned medium, which could not be obtained using static culture [29]. Cortiella *et al* used the RWV for repopulating various natural and synthetic ECM scaffolds with ESCs, including decellularized lung scaffolds [7]. While this study demonstrated enhanced cell differentiation and viability of ESCs on decellularized scaffolds compared to other ECM scaffolds in the RWV, the authors focused on the role of different ECM scaffolds (and not on RWV growth conditions) as identical static controls were not included. Therefore, it remained unclear whether the standard RWV culture conditions (~20 RPM) that enhanced cellular differentiation and growth in previous reports [27–29,31], could be utilized to improve recellularization efficiency and stem cell differentiation in decellularized lung scaffolds.

In this study, we examined whether the use of the RWV would (i) enhance recellularization efficacy using two relevant mouse cell types, i.e. bone marrow derived MSCs and alveolar type II epithelial cells (C10), and (ii) affect differentiation of the inoculated MSCs in the decellularized lungs.

## Materials and Methods

### Cells, media and growth conditions

MSCs from bone marrow of adult C57BL/6 mice were obtained from the NCRR/NIH Center for Preparation and Distribution of Adult Stem cells at Texas A&M University [32]. C10 cells are a non-transformed alveolar type II epithelial cell line derived from normal BALB/c mouse lungs [33], and were kindly provided by Matthew Poynter (University of Vermont). MSCs were cultured in Iscove’s Modification of Dulbecco’s Medium (IMDM, basal medium), according to previous reports using this cell type [2,3,15,18], supplemented with 10% fetal bovine serum (Invitrogen), 10% horse serum (Invitrogen), 2 mM L-glutamine (Sigma), 100 μg/mL primocin (InvivoGen), and 100 U/mL penicillin and 100 μg/mL streptomycin (Sigma). MSCs were grown in T175 aerated flasks to 70% confluency and were used up to passage 7. C10 cells were cultured in a basal cell culture medium GTSF-2 [34], based on previous reports on alveolar epithelial cell culturing in the RWV bioreactor [27,28], modified by replacing fungizone with 100 μg/mL primocin and supplementing with 1.5 g/L NaHCO_3_ to support growth in a 5% CO_2_ incubator at 37°C.

### Decellularization of lungs

Heart-lung blocs of adult male and female C57BL/6 mice (8–24 weeks, Jackson Laboratories) were decellularized using an SDS-Triton based detergent approach as described previously [2,6]. This study was approved by the University of Vermont (UVM) Institutional Animal Care and Use Committee (IACUC), and animals were maintained at UVM in accordance with IACUC standards and review (UVM IACUC 11–003 “Bioengineering New Lungs from Cadaveric Scaffolds”). Euthanasia was performed under standards of the Association for Assessment and Accreditation of Laboratory Animal Care International (AAALAC) and UVM IACUC using lethal intraperitoneal injection of sodium pentobarbital followed by removal of the heart lung bloc. Animals experienced only momentary distress during the injection.

### Recellularization of decellularized lungs in static and RWV bioreactor conditions

**Fig 1:**
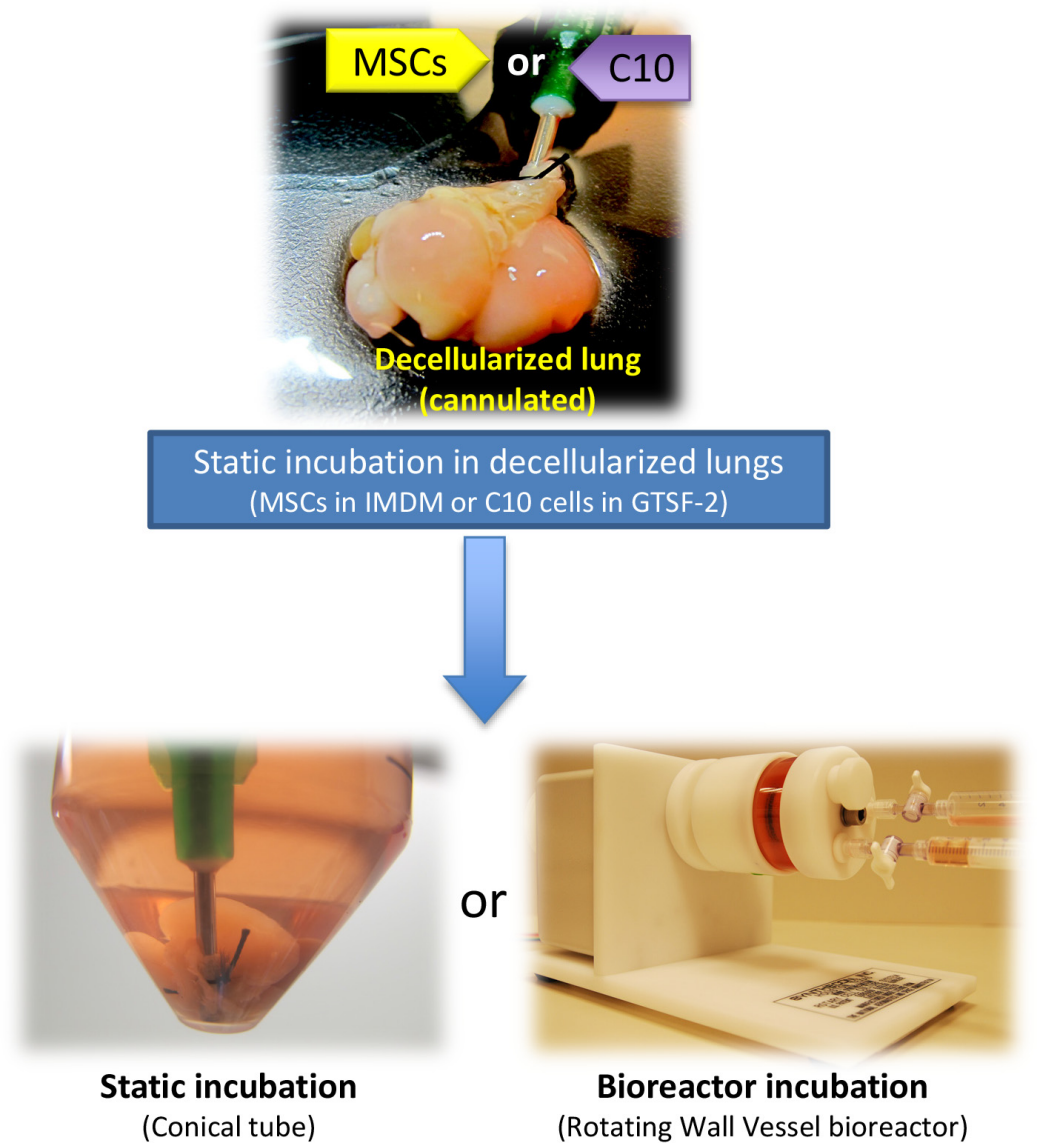
Overview of experimental set-up used to recellularize decellularized lung scaffolds with MSCs or C10 cells in static and bioreactor conditions. MSCs or C10 cells were introduced in the decellularized lung scaffolds through the cannulated trachea. Next, lungs were statically incubated for 4 days, regardless of the subsequent test condition. Culture medium for MSCs was IMDM and for C10 cells GTSF-2. Different time points were tested to assess recellularization with MSCs (3, 10, 24 days) or C10 cells (7, 10 days) in static or bioreactor conditions.

An overview of the experimental set-up is provided in **Fig 1**. For recellularization in both static and RWV conditions, 4 x 10^6^ MSCs or C10 cells grown as monolayers were suspended in 3 mL IMDM or GTSF-2 medium, respectively and injected in the decellularized lung scaffold through the cannulated trachea. The cell number injected per lung is in the range of previous reports [2,15,18]. Any liquid that was not retained in the lungs upon the first injection was re-injected up to five times, until more than 99% of the initially added cells remained in the scaffolds. Each seeded heart-lung bloc was transferred to a 50 mL conical tube containing 15 mL of IMDM (for MSCs) or GTSF-2 (for C10 cells) medium and incubated in a 37°C, 5% CO_2_ incubator. Lungs for static recellularization were kept in these conditions for the duration of the experiment, and medium was changed every 2–3 days. Lungs for RWV bioreactor recellularization were maintained statically for 4 days to allow adequate initial adherence of cells to the matrix, after which the cannula was removed and the heart-lung bloc subsequently transferred to a 50 mL RWV bioreactor. The need for static incubation prior to bioreactor incubation was determined empirically, since immediate transfer of lungs to the bioreactor precluded effective cell attachment. A rotation speed of 20 rpm was adopted to keep the lungs in suspension culture, and lungs were incubated for 3, 10, and 24 additional days in the RWV (total incubation of 7, 14, and 28 days respectively) for MSCs, or 7 and 10 additional days in the RWV for C10 cells (total incubation of 11 and 14 days respectively). Bioreactors were inspected daily for potential formation of air bubbles, removed if necessary and culture media was changed weekly given the larger bioreactor volume (50 mL) compared to static conditions (15 mL).

### Fixation of static and RWV-recellularized lungs for mRNA expression and immunohistochemistry

Per heart-lung bloc, one lung was assessed for total RNA contents and for mRNA expression by quantitative real time PCR (qRT-PCR), and the other lung was fixed for immunohistochemistry. The lungs for RNA analysis were incubated overnight at 4°C in RNA Protect (Qiagen) and were then stored at -80°C until processed for RNA extraction and purification. The lungs for immunohistochemistry were fixed using 4% paraformaldehyde (PFA) (Electron Microscopy Services) for 30 min and were stored at 4°C in sterile PBS until further processing.

### mRNA expression analysis

The total RNA of fixed lung samples was isolated using TRIzol reagent (Life Technologies) followed by purification with the RNeasy kit (Qiagen), according to the manufacturer’s instructions. RNA quantity and quality was assessed using a Nanodrop spectrophotometer. RNA was converted to cDNA using the Monsterscript 1^st^-strand cDNA synthesis kit (Epicenter Biotechnologies). Quantitect SYBR Green Master mix (Qiagen) was used to assess differential gene expression with qRT-PCR, according to the manufacturer’s protocol. Potential differentiation of MSCs was assessed through expression of markers typically (but not solely) expressed by (i) different types of lung epithelial cells (tight junctional marker ZO-1, water channel marker AQP5, and SPD), specific lung cell populations (CCSP and Scgb3a2 for Club cells—formerly named Clara cells; SPC, SPB, SPA for type II alveolar epithelial cells; MUC5AC and Spdef for goblet cells; Trp63 for basal cells) [35], early lung differentiation markers (TTF-1 or NKX2.1, and FoxJ1) [35], (ii) adipocytes (adiponectin), (iii) osteocytes (osteopontin), (iv) cartilage (Col2a1), (v) fibroblasts and other mesenchymal cells (FAP, Fsp1, Col1a1, FN, ∞-SMA), (vi) a cytokine (TGF-β), (vii) tumor-associated MSCs (stanniocalcin, MMP3) [36,37], and (viii) multipotent cells (endoglin, Sca-1, CD106) (abbreviations and primers are defined in **S1 Table**). Primer specificity was tested on DNA from MSCs grown as monolayers (extracted using the DNeasy kit, Qiagen) and on cDNA from (i) MSCs grown as monolayers and (ii) mouse lungs (pooled RNA from 25 lungs of 25 week old male mice, Amsbio). The qRT-PCR reactions were performed using an Eppendorf Mastercycler EP RealPlex 2S system. A melting curve was run at the end of each reaction to test for the presence of a single PCR product. The qRT-PCR reaction product was run on a 3% agarose gel in the presence of a low molecular weight DNA ladder (New England BioLabs), to assess for potential non-specific binding and primer dimerization. C_T_ values were exported using the Eppendorf Database tool, whereafter the delta delta C_T_ method [38] was adopted to determine relative gene expression between different test conditions. An average of six housekeeping genes was used for normalization (β-actin, GAPDH, Yhwaz, Sdha, Tbp, and Pkg) [39,40]^,^ [41]. The gene expression of MSCs grown under static or bioreactor conditions was compared to the expression of MSCs grown as conventional monolayers.

### Histology

Following PFA fixation (see above), recellularized lungs were paraffin-embedded, and 5 μm slices were mounted on positively charged glass slides. Paraffin-embedded normal mouse lung slices (Amsbio) were included as a histology control and were processed the same way. Staining with hematoxylin/eosin was performed using standard protocols [2]. Alizarin red staining was performed to assess potential differentiation of MSCs along the osteoblastic lineage in the different test conditions, and was done using standard protocols [2]. For Alizarin red staining, a negative control (decellularized lungs without cells) and positive control (MSC monolayers differentiated along the osteoblastic lineage) was included as well. For this positive Alizarin red control, MSC monolayers were exposed to osteogenic differentiation medium, comprised of IMDM supplemented with 1 nM dexamethasone, 50 μM L-ascorbic acid-2 phosphate, and 20 mM β-glycerol phosphate, for 3 weeks [42], where after they were processed for immunohistochemistry as described above. Stained slices were imaged by standard bright field microscopy (100x and 400x) (Zeiss AxioVert A1), and at least 10 regions in each of 5 tissue slices per sample were analyzed.

### Immunofluorescence staining and imaging

Paraffin-embedded slices from recellularized lung scaffolds and control mouse lung slices (Amsbio) were deparaffinized by sequential incubation in three baths (150 mL/bath) of xylene for 5 min, one bath of 50% xylene 50% absolute ethanol for 2 min, two baths of absolute ethanol for 2 min, one bath of 95% ethanol for 2 min, one bath of 70% ethanol for 2 min, followed by rinsing with MilliQ water. Then, antigen retrieval was performed by heating tissue slices in sodium citrate buffer (10 mM sodium citrate, 0.05% Tween 20, pH 6.0) (Dako) at 100°C for 20 min and cooling to room temperature prior to immunofluorescence staining. Tissue sections were stained with specific antibodies as described previously [27]. The following primary antibodies were used: CC10 or uteroglobin (Abcam, ab40873, 1:1600), osteopontin (Abcam, ab8448, 1:100), collagen I (Abcam, ab292, 1:100), Fsp1 (Abcam, ab41532, 1:200), Annexin V (Abcam, ab14196, 1:500), and PCNA (Abcam, ab29, 1:1000). Secondary antibodies used in this study were: goat anti-mouse Alexa Fluor 488 or 555 (Invitrogen), goat anti-rabbit Alexa Fluor 488 or 555 (Invitrogen) at a 1:500 dilution. Cell nuclei were stained with DAPI (Prolong Gold with DAPI mounting solution, Invitrogen). Stained tissue sections were imaged using a Zeiss LSM 510 Duo laser scanning microscope or Zeiss Axiovert A1. Images were acquired using a Plan-Neofluar 40x or 63x objective and were analyzed with the Zeiss LSM software package or Zen Lite. Axiovision 4.7 or Zen 2011 softwares from Carl Zeiss were used to further process collected images.

### Statistical analysis

All studies were conducted at least in biological triplicate. Statistical significance (α = 0.05) was determined using a two-sample Student’s t-test on the biological replicates. To determine the suitability of the housekeeping genes and their average for normalization of the qRT-PCR data, the coefficient of variation was calculated. Since the coefficient of variation for target, housekeeping genes and the average of housekeeping genes was comparable (**S1 Fig**), the adopted normalization approach did not influence the gene expression data.

## Results

### Enhanced recellularization of decellularized lung scaffolds with MSCs in bioreactor versus static conditions

**Fig 2:**
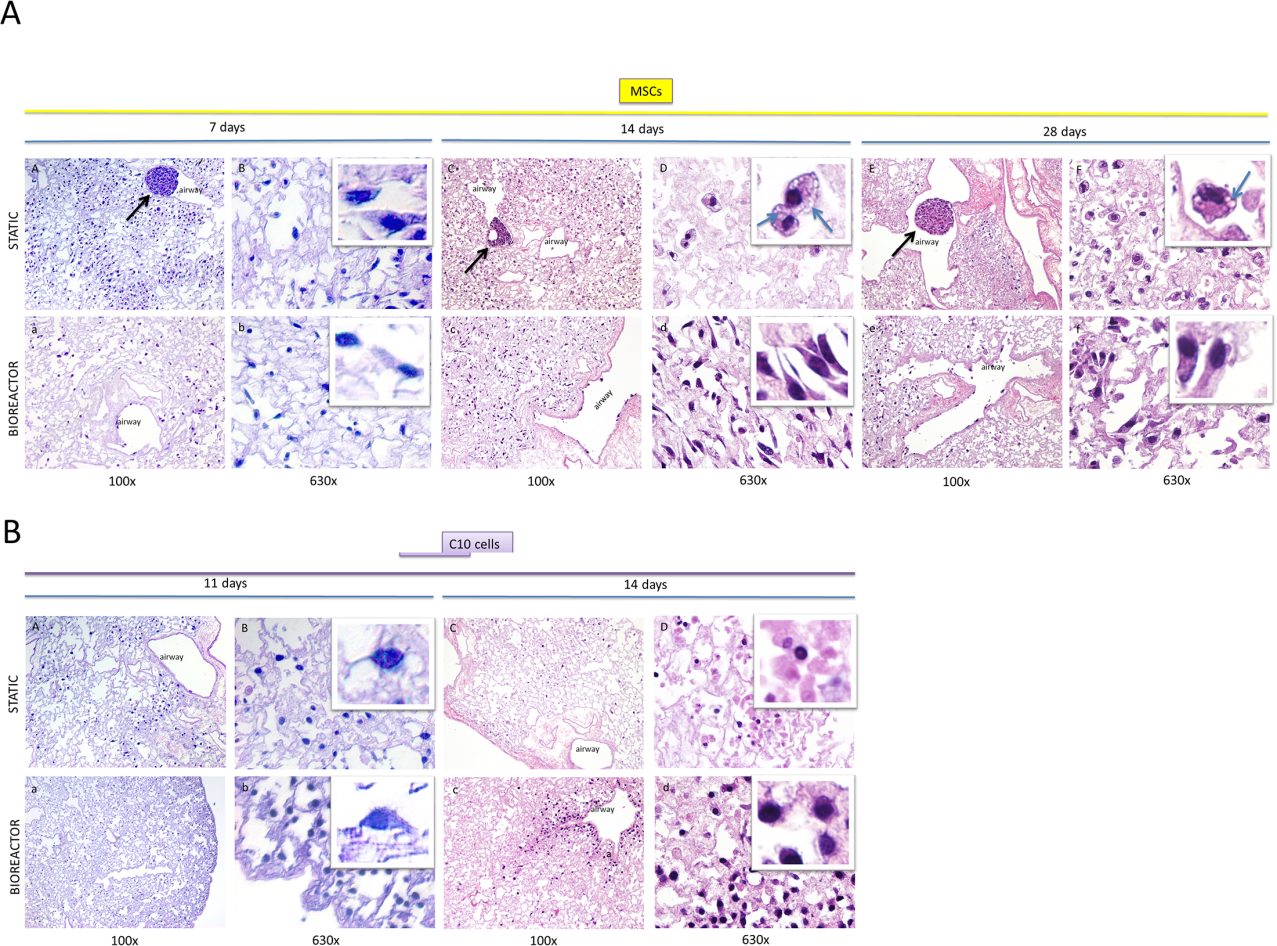
Hematoxylin-eosin staining of decellularized lungs recellularized with (A) MSCs in static (panels A to F) and bioreactor (panels a to f) conditions for 7, 14, and 28 days, and (B) with C10 cells in static (panels A to D) or bioreactor (panels a to d) conditions for 11 and 14 days. For each condition, a low (100x) and high magnification (630x) are shown (e.g., A is low magnification, B is high magnification). Insets are included to show phenotypes at the single cell level. Black arrows point to MSC cell aggregation observed in static recellularization conditions. Blue arrows point to cytoplasmic vacuoles indicative of cell stress. Airways are labelled. For each condition, images are representative of the entire lung, with the exception of panels AA and AB, which reflect a region with high cell density whereas some regions were devoid of cells (not shown).

**Fig 3:**
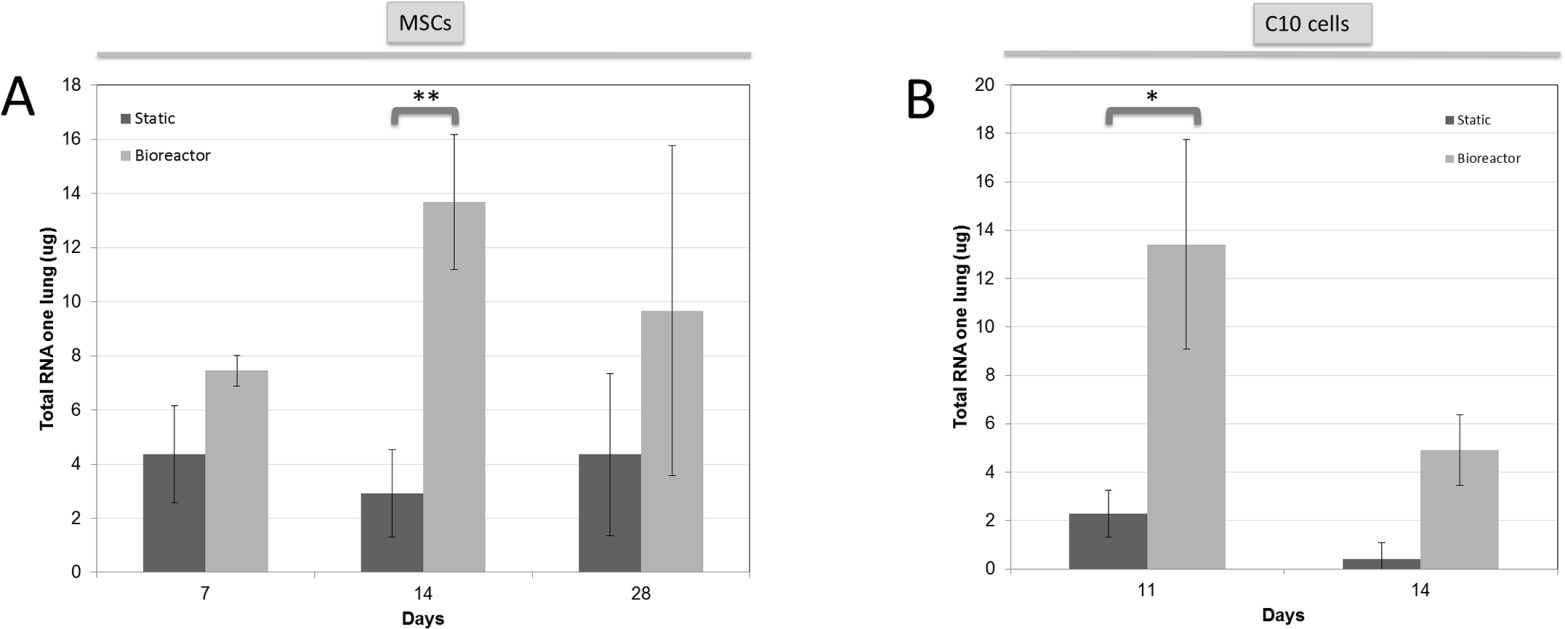
Total RNA levels of decellularized lungs recellularized with MSCs (A) or C10 cells (B) in static versus bioreactor conditions. For each condition, mean RNA levels +/- standard deviation for the left lung is presented. * p < 0.05, ** p < 0.01.

Decellularized lung scaffolds recellularized with MSCs for a total of 14 days had more abundant cells in the bioreactor as compared to static conditions (**Fig 2AD versus Fig 2Ad**). In accordance with this observation, the total RNA quantity for lungs recellularized with MSCs in bioreactor conditions on day 14 was on average 3.8 times higher as compared to static recellularization (p < 0.01) (**Fig 3A**). No significant differences in cell repopulation were observed at 7 and 28 days of MSC culture based on histology and total RNA levels (**Figs 2 and 3**).

MSCs were non-uniformly distributed after 7 days of static recellularization, with regions containing high cell numbers (**Fig 2AA and 2ABB**) and regions devoid of cells (not shown). In contrast, cells were more homogeneously distributed throughout the scaffold in the bioreactor culture (**Fig 2Aa and 2Ab**). At 14 and 28 days, fairly homogenous recellularization was generally observed for both static and bioreactor conditions, but both test conditions had regions in which cells were absent. In both static and bioreactor conditions, MSCs were present predominantly in the alveoli, but some recellularization of large and small airways was observed as well.

At 7 days of culture, both test conditions showed similarities in MSC morphologies, varying from rounder to elongated phenotypes. After 14 days of culture, statically recellularized lungs mostly contained round cells while bioreactor-recellularized lungs contained MSCs with rounder to spindle-shaped morphologies in airways and alveoli (**Fig 2AD and 2Ad**). At day 28, the number of round cells containing cytoplasmic vacuoles (indicative of cell stress) was enhanced in both conditions (compared to day 7, 14), however, there continued to be more elongated cells without cytoplasmic vacuoles in bioreactor-recellularized lungs (**Fig 2AF and 2Af**). Also at 14 days of culture, MSCs in static conditions displayed enhanced formation of cytoplasmic vacuoles (**Fig 2AD**, blue arrows).

Interestingly, formation of scattered MSC clusters was observed in static but not bioreactor conditions at the three studied time points (**Fig 2AA, 2AC and 2AE**). The larger cell clusters were mostly found in small airways but smaller aggregates were also observed in alveolar regions.

### Enhanced recellularization of decellularized lung scaffolds with C10 cells in bioreactor versus static conditions

Recellularization in static and bioreactor conditions with C10 cells was initially studied for 14 days based on the results obtained with MSCs. In static conditions, whole lungs were poorly recellularized, abundant debris and ghost cells were observed and RNA levels were low (**Fig 2BC and 2BD; Fig 3B**). In contrast, at this same timepoint, recellularization under bioreactor conditions resulted in intact cells that were primarily located at the periphery of the scaffolds and surrounding larger airways (**Fig 2Bc**), and approximately 12-fold higher RNA recovery was observed (p < 0.05) (**Fig 3B**). Still, many regions were populated with ghost cells and debris (**Fig 2Bc**), and therefore a shorter incubation time was assessed. At 11 days, limited cell debris was observed in both test conditions, cells were non-uniformly distributed and mostly recellularized the alveoli (**Fig 2BA and 2BB**for static and **Fig 2Ba and 2Bb**for bioreactor). An increase in RNA recovery was obtained from lungs recellularized with C10 cells for this shorter time frame in both test conditions, with bioreactor lungs containing about 6 times more RNA than static lungs (p = 0.07) (**Fig 3B**). For recellularization with either MSCs or C10 cells, lungs collapsed and shrank over time, both in static and bioreactor conditions.

### MSCs and C10 cells exhibited less apoptosis and higher cell proliferation in bioreactor versus static recellularization conditions

**Fig 4:**
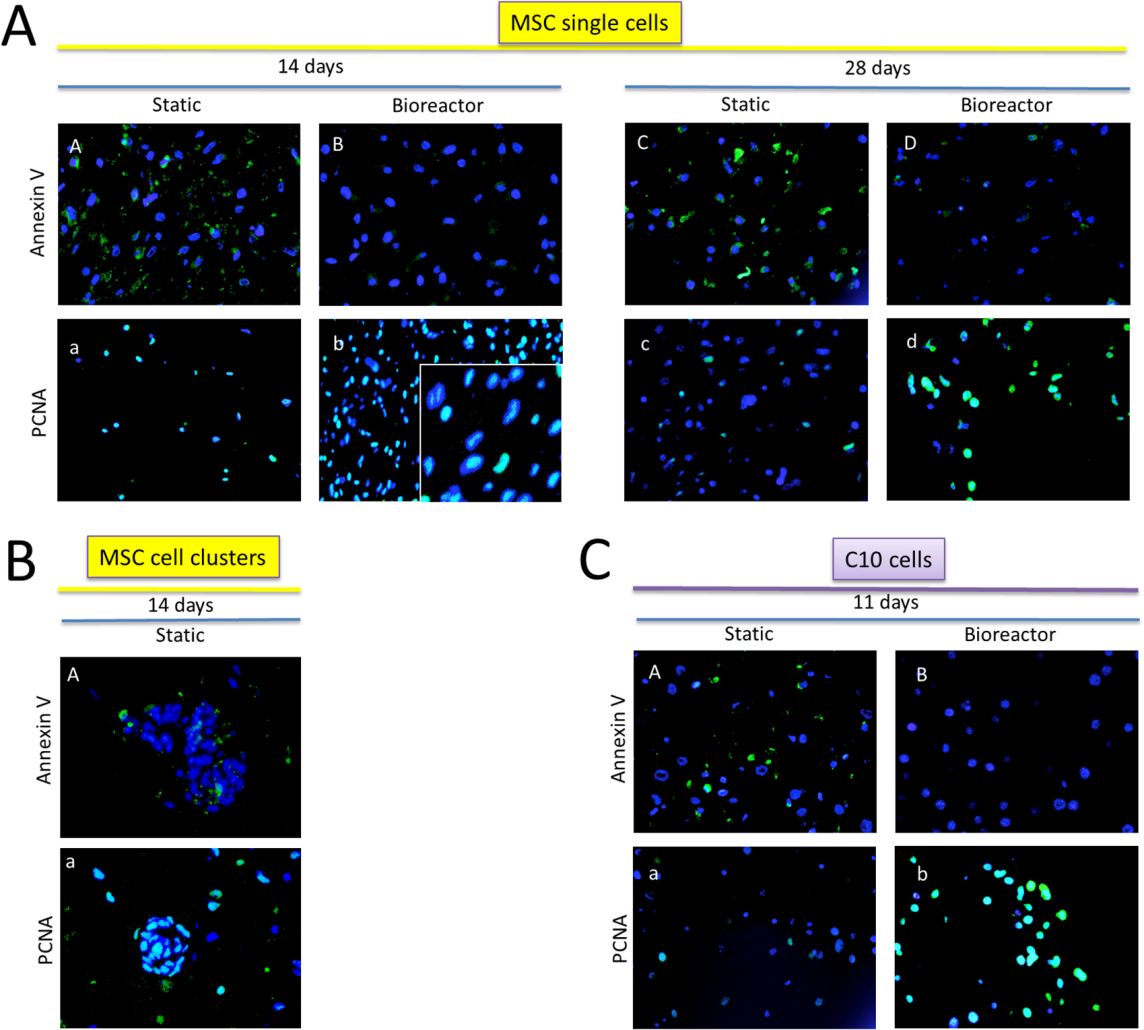
Annexin V and PCNA staining of decellularized lung scaffolds recellularized with (A) MSCs in static versus bioreactor conditions for 14 (panels A, B for annexin V, and a, b for PCNA) and 28 days (panels C, D for annexin V, and c, d for PCNA) (single cells), (B) MSC cell clusters in static conditions at 14 days (panel A for annexin V, and a for PCNA), (C) C10 cells in static (panel A for annexin V, a for PCNA) versus bioreactor (panel B for annexin V, b for PCNA) conditions for 11 days. An inset in Fig 4Ab with higher magnification is shown to demonstrate that a majority of the cells stained positive for PCNA. Cell nuclei are labeled in blue; marker of interest is labeled in green. Magnifications are 400x. Overlap of cell nucleus and marker of interest can generate green or white color. For each condition, images are representative of the entire lung.

In agreement with the H&E observations for MSCs at day 14, qualitatively more cells stained positive for the apoptosis marker annexin V in static compared to bioreactor conditions (**Fig 4AA versus Fig 4AB**). Cells within cell clusters (static) showed a similar staining pattern as single MSCs (**Fig 4BA**). After 28 days of culture, most MSCs in static conditions stained positive for annexin V, while fewer cells stained positive in bioreactor conditions (**Fig 4AC versus Fig 4AD**). Similarly, static recellularized lungs contained a high number of annexin V-positive C10 cells and cell debris at day 11, while the bioreactor condition showed cells that were mostly negative for annexin V (**Fig 4CA versus Fig 4CB**).

Staining for the cell proliferation marker PCNA indicated that a majority of the MSCs were actively proliferating at 14 days in both static and bioreactor conditions (**Fig 4Aa and 4Ab**). Cells within cell clusters (static) also stained positive for PCNA (**Fig 4Ba**). At day 28, a limited number of cells were PCNA positive in static conditions, while most bioreactor-grown MSCs stained positive for this marker (**Fig 4Ac and 4Ad**). With regard to C10 cells, statically recellularized lungs showed few regions with cells positive for PCNA, while a majority of the cells in bioreactor-recellularized lungs were PCNA positive (**Fig 4Ca and 4Cb**).

### Differentiation of MSCs grown on decellularized lung scaffolds recellularized in static and bioreactor conditions

Next, the gene expression and phenotypic analysis of MSCs was determined when grown on decellularized lung scaffolds in static and bioreactor conditions. MSCs were chosen for further analysis since these stem cells (i) hold potential for *ex vivo* generation of lung tissue, and (ii) remained viable for longer term compared to C10 cells in the decellularized lung scaffolds. Main differences in gene expression between static and bioreactor conditions at day 14 included the downregulation of Col1a1 (Collagen I alpha I) and upregulation of endoglin and CD106 for statically recellularized lungs, but not for bioreactor lungs, compared to MSC monolayers (**Table 1**). For the bioreactor growth condition, we observed upregulation of genes encoding Fsp1 (fibroblast specific protein), TGF-β and adiponectin, as compared to the MSC monolayer control (**Table 1**).

**Table 1:** Relative expression of target genes in MSCs grown on decellularized lung scaffolds in static and bioreactor conditions as compared to monolayers.

	7 days		14 days	
	Static IMDM	Bioreactor IMDM	Static IMDM	Bioreactor IMDM
α-SMA				*9*.*09* *
Adiponectin		10.95***		31.53***
Aqp5	**3.35** *	1.45**	2.83***	1.99**
CCSP		**16.50** *	41.01***	37.41***
CD106	∞	∞	**34.88** *	
Col 1a1	*4*.*72* ***	*1*.*81* **	*20*.*09* **	
Col 2a1			*7*.*79* ***	*9*.*72* ***
Endoglin			12.37**	
FAP		26.01***	8.23**	119.87***
FN1				
FoxJ1	∞	∞	∞	∞
FSP1				**4.73** *
MMP3	∞	∞	∞	∞
MUC5AC	∞	∞	∞	∞
Osteopontin	56.02**	27.09***	108.20***	50.28***
Sca-1				
Scgb3a2	∞	∞	∞	∞
SPA	Ф	Ф	Ф	Ф
SPB	Ф	Ф	Ф	Ф
SPC				
SPD	∞	∞	∞	∞
Spdef	∞	∞	∞	∞
Stanniocalcin	∞	∞	∞	∞
TGF-β				4.06**
Trp63	∞	∞	∞	∞
TTF1				
Vimentin				
ZO-1				

* P < 0.08

** P < 0.05

*** P < 0.01

Bold = upregulated compared to MSC monolayer; Italic = downregulated compared to MSC monolayer; White empty cell: P > 0.08 and/or fold-change < 1.5; While cell with ∞: Present in ML, absent in ≥ 50% of samples; White cell with Ф: Not expressed in ML and sample, but expressed in lung.

In both static and bioreactor conditions at day 14, MSCs significantly upregulated the expression of AQP5 (Aquaporin 5), CCSP (Club Cell Secretion Protein), osteopontin and FAP (Fibroblast activation protein), and downregulated the expression of Col2a1 (Collagen 2 alpha 1) compared to monolayer controls (**Table 1**). Interestingly, most of the gene expression changes at 14 days of culture were reflected in the 7-day cultures (**Table 1**). None of the other tested genes showed differential expression in either condition.

**Fig 5:**
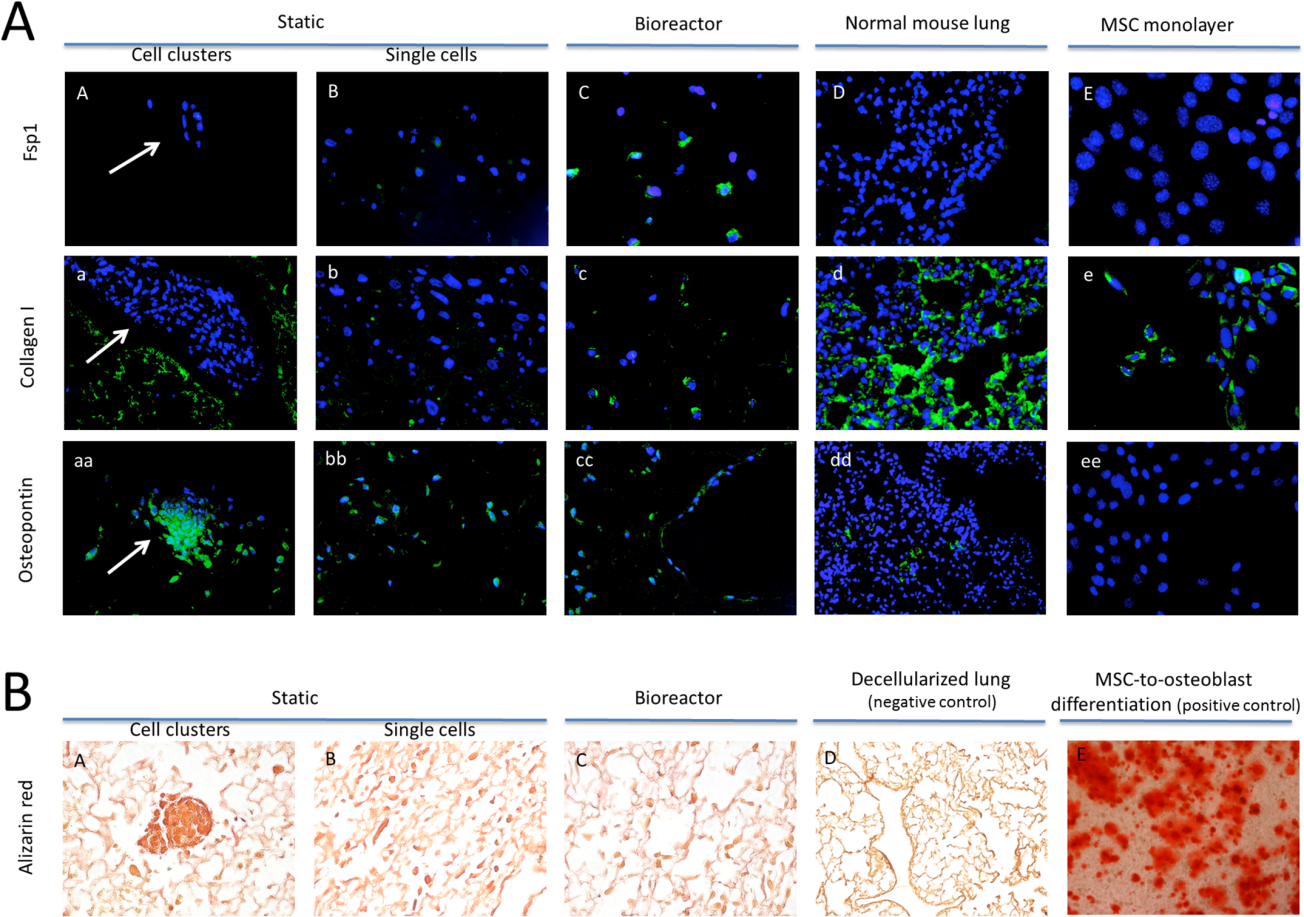
A: Immunohistochemical profiling of decellularized lung scaffolds recellularized with MSCs in static and bioreactor conditions for 14 days. Profiling of whole normal mouse lung tissue and MSC monolayers was performed as well. Cell nuclei are labeled in blue; markers of interest are labeled in green and are Fsp1 (panel A to E), collagen I (panel a to e), and osteopontin (panel aa to ee). White arrows point to multilayered cell aggregates, observed in static recellularization conditions and for this test condition profiling for both aggregates (A, a, aa) and single cells (B, b, bb) is presented. Since collagen I-positive cells showed higher signal intensity compared to that of the collagen I-positive scaffold, the background scaffold signal in Fig 5Ab and 5Ac was removed for clarity. To demonstrate the collagen I-positive scaffolds, the background signal is only shown in Fig 5Aa. Magnifications are 400x or 630x. B: Alizarin red staining of decellularized lung scaffolds recellularized with MSCs in static (panels A, B) and bioreactor (panel C) conditions for 14 days. As a negative control, decellularized lungs that were not seeded with cells are presented (panel D). MSC monolayers differentiated along the osteoblastic lineage are included as positive control (panel E). Magnification is 200x or 630x. For each condition, images are representative of the entire lung.

In agreement with the gene expression data, a high number of MSCs stained positive for the lung fibroblast marker Fsp1 in bioreactor conditions while only a few cells were positive in static conditions on day 14 (**Fig 5AC, 5AB and 5AA**). MSC monolayers (**Fig 5AE**) and normal whole mouse lung controls (**Fig 5AD**) stained negative for Fsp1.

Higher numbers of MSCs stained positive for collagen I in the bioreactor compared to statically-recellularized lungs (**Fig 5Ab and 5Ac**), which corresponds to the gene expression results. However, fewer MSCs stained positive for collagen I in either bioreactor or static-recellularized lungs compared to MSC monolayers (**Fig 5Ac, 5Ab and 5Ae**). The decellularized lung matrix stained positive for collagen I in most regions, regardless of the presence of MSCs (**Fig 5Aa**) and normal mouse lung tissue was also collagen I-positive (**Fig 5Ad**).

Osteopontin was found highly expressed in MSCs grown on decellularized lung scaffolds in both static and bioreactor conditions (**Fig 5Aaa, 5Abb and 5Acc**), while MSC monolayers were negative (**Fig 5Aee**) and normal control lungs showed occasional expression (**Fig 5Add**). To further assess whether MSCs differentiated along an osteogenic lineage, alizarin red staining was performed. Despite the ability to differentiate MSCs into alizarin red-positive cells in routine tissue culture (positive control) (**Fig 5BE**), minimal numbers of alizarin red-positive cells were observed in most of the recellularized lung tissues from either static or bioreactor conditions (**Fig 5BA, 5BB and 5BC**).

MSCs grown as conventional monolayers, or on decellularized lung scaffolds in static and bioreactor conditions did not positively immunostain for CC10 (data not shown), despite strong induction of this marker at the gene expression level on the scaffolds (**Table 1**).

The multilayered cell clusters observed in static recellularization conditions tested positive for osteopontin (but negative for alizarin red) and showed few or no cells that stained positive for collagen I and Fsp1 (**Fig 5AA, 5Aa and 5Aaa; Fig 5BA**). Of note, positive staining of the decellularized lung matrix for collagen I is shown in **Fig 5Aa**. Therefore, the cell clusters seemed to show similar staining patterns as single MSCs.

## Discussion

A promising approach for *ex vivo* lung engineering is the repopulation of decellularized lung scaffolds with autologous stem and/or progenitor cells derived from the actual transplant recipient. The decellularized scaffolds also provide a novel tool for studying cell-matrix interactions and other aspects of lung biology. While decellularized lung scaffolds derived from rodents, sheep, pigs, primates, and humans have been repopulated with a variety of different cell types [2–10,12–15,17,18,43], improved culture techniques are needed to enhance the full recellularization of the lung surface area with viable, differentiated cells. In addition, as critical cell binding epitopes on the ECM proteins remaining in the scaffold may be affected by the decellularization process [2,3], restoring the normal ECM content and function of the decellularized scaffolds is of importance to support cell growth and differentiation.

In this study, we demonstrated that recellularization efficiency (i.e. number of cells adhering to scaffolds) and viability were improved when decellularized lung scaffolds were cultured in a dynamic suspension bioreactor system (RWV) as specifically compared to static conditions. We speculate that the biomechanical force of fluid shear in the RWV and associated mass transfer result in differential oxygenation, nutrient availability, waste dispersal, some or all of which may have contributed directly or indirectly towards our phenotypic and molecular genetic observations [22]. In agreement with our results, growth stimulation of progenitor cells in the RWV bioreactor has been reported previously [19,44]. Cortiella *et al* previously demonstrated that decellularized lungs repopulated with ESCs in the RWV retained more viable cells and induced higher levels of apparent lung-specific lineages in response to defined differentiation agents, compared to other synthetic scaffolds and 2-D ESC monolayers [7].

In our study, the enhanced attachment of MSCs to the decellularized scaffolds in the bioreactor could, in part, be potentially explained by induced TGF-β expression, which has been shown to enhance attachment of MSCs to collagen I through induction of integrin subunits [45]. The well-spread polygonal cell shape predominantly observed in bioreactor conditions is an indicator of healthy MSCs [46,47]. On the other hand, this characteristic cell shape could also imply a lower multipotentiality and higher commitment towards differentiation as compared to the rounder phenotype in static conditions [48–50]. In line with this hypothesis is (i) the induced expression of the multipotency markers endoglin and CD106 in static but not bioreactor conditions, and (ii) the absence of MSC cell clusters in bioreactor growth conditions, which could possibly be a characteristic of multilineage-differentiating stress-enduring (Muse) cells that have the ability to differentiate into endoderm, ectoderm and mesoderm *in vitro* and *in vivo* [51,52]. Alternatively, it is possible that the stacked cell clusters observed in static conditions could reflect an abnormal cell phenotype.

Our collective data suggest that a portion of the bioreactor-cultured MSCs may have differentiated into fibroblast-like cells (expression of Fsp1, FAP, osteopontin, collagen I). Fsp1 is specifically expressed in fibroblasts but at very low levels in epithelium and MSCs [53–55] and has been correlated with collagen deposition and lung remodeling in bleomycin-treated mice [54]. Similarly, FAP and osteopontin are involved in lung tissue remodeling [56,57]. Since fluid shear triggers tissue remodeling and extracellular matrix deposition [58,59], enhanced fluid shear levels in the bioreactor could potentially have caused the enhanced generation of fibroblasts. Hence, the induction of fibroblast markers in bioreactor culture conditions could contribute to the regeneration and/or remodeling of decellularized lung scaffolds. Specifically, since decellularized lung scaffolds are depleted of key ECM proteins that could play a role in cell growth and differentiation [2,5,8], we speculate that potential restoration of the native ECM scaffolds by fibroblast-like cells could be beneficial for *ex vivo* lung tissue generation. In a similar fashion, mESCs recellularizing the lung scaffolds in the RWV bioreactor secreted ECM components that were depleted in the acellular lung [7].

Growth of MSCs on decellularized lung scaffolds in both static and bioreactor test conditions in basal medium induced the expression of mRNAs encoding two markers that are expressed in lung epithelial cells (as well as other cell types), AQP5 and CCSP. Recent studies suggest basal expression of lung epithelial markers in some preparations of MSCs, and in response to lung differentiation media [13,60,61]. However, CCSP expression could not be confirmed at the protein level in our study. Discrepancies between qRT-PCR and immunofluorescence results have been reported previously for MSCs on decellularized lung scaffolds, and could potentially be explained by differences in sensitivity of both methods [13] or by potential posttranscriptional modifications [62]. An alternative explanation is the inability of the growth conditions used in this study to support phenotypic expression of these markers.

In a similar fashion as for MSCs, bioreactor conditions gave rise to lung scaffolds populated with a higher number of viable C10 cells as compared to static conditions. Previous studies described abundant apoptosis of C10 cells when grown in decellularized whole lungs statically for 7 to 14 days, or during continuous vascular perfusion [3,10,18]. When C10 cells were grown on decellularized lung slices, only limited apoptosis was observed up to 28 days of culture [15]. Collectively, these data indicate that C10 cells could be sensitive to the growth conditions inside whole decellularized lungs. We and others have observed shrinking and collapsing (atelectasis) of the scaffolds as a function of time [7], which could result in oxygen and nutrient limitation and waste product accumulation. Therefore, the continuous movement of the lung scaffolds in the medium during recellularization in the RWV bioreactor, which results in differential oxygenation, nutrient provision and waste dispersal [22], may have provided more optimal culture conditions for this particular cell type as compared to static culture.

In conclusion, we demonstrated that the RWV bioreactor confers advantages for recellularization of decellularized lung scaffolds, with regards to cell growth, cell health, and differentiation as compared to static conditions. Since MSCs have been suggested to serve as stroma for repopulating decellularized scaffolds with other cell types (such as endogenous progenitors and induced pluripotent stem cells) [63], further investigations into whether the enhanced expression of genes involved in ECM remodeling in the RWV bioreactor resulted in reconstituting and/or remodeling of the native ECM scaffold is of interest. Furthermore, this study could have downstream implications for studying tissue homeostasis and identifying the underlying factors that contribute to the transition of normal to fibrotic lung phenotypes.

## Supporting Information

S1 Fig Coefficient of variation for target genes, housekeeping genes and average of housekeeping genes for qRT-PCR gene expression data at 7 days (A) and 14 days (B) of culture in the different test conditions. (TIF)

S1 Table Primers used for qRT-PCR analysis. (DOCX)
