# Supplementary figures and images for: Recellularization of Decellularized Lung Scaffolds Is Enhanced by Dynamic Suspension Culture
Source: PLoS One. 2015 May 11;10(5):e0126846. doi: 10.1371/journal.pone.0126846 (PMC4427280; doi:10.1371/journal.pone.0126846)

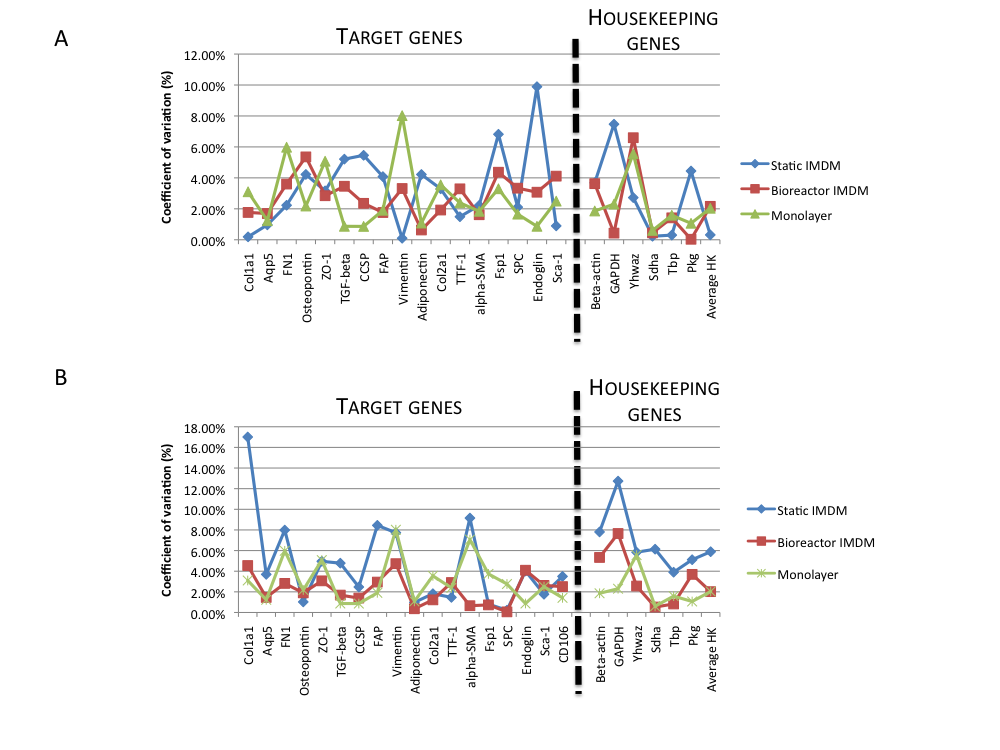

Supplement: S1 Fig — (TIF) [file pone.0126846.s001.tif]
