# Supplementary material for: Recellularization of Decellularized Lung Scaffolds Is Enhanced by Dynamic Suspension Culture
Source: PLoS One. 2015 May 11;10(5):e0126846. doi: 10.1371/journal.pone.0126846 (PMC4427280; doi:10.1371/journal.pone.0126846)
Supplement: S1 Table — (DOCX) [file pone.0126846.s002.docx]

**Table S1. Primers used for qRT-PCR analysis**

| **Gene** | **Abbreviation** | **Forward primer (5’ – 3’)** | **Reverse primer (5’ – 3’)** | **Amplicon size (bp)** | **Category** | **Reference** |
| --- | --- | --- | --- | --- | --- | --- |
| α-SMA | Smooth muscle actin | CCTGAAGAGCATCCGACAC | GAGTCCAGCACAATACCAG | 173 | MS | (Daly *et al.*, 2012) |
| AdipoQ | Adiponectin | GCAGGCATCCCAGGACATC | GCGATACACATAAGCGGCTTCT | 216 | AC | (Daly *et al.*, 2012) |
| Aqp5 | Aquaporin 5 | CTGTACTGGTTGGCGCCAG | TCCTACCCAGAAGACCCA | 315 | Lung | (Daly *et al.*, 2012) |
| B-actin | B-actin | GTGGGCCGCTCTAGGCACCAA | CTCTTTGATGTCACGCACGATTTC | 540 | HK | (Daly *et al.*, 2012) |
| CCSP | Clara Cell Secretion Protein | ATGAAGATCGCCATCACAATCAC | GGATGCCACATAACCAGACTCT | 135 | CS | This study |
| CD106 | Cluster of differentiation 106 | CATGACAAAACTCAGGAGCCA | TGCCTTCACTTTTGGGGTATCT | 98 | MP | This study |
| Col1a1 | Collagen 1 alpha 1 | CCGCCGATGTCGCTATCCAG | GTGACTCGTGCAGCCGTCCAC | 221 | CT | (Daly *et al.*, 2012) |
| Col2a1 | Collagen 2 alpha 1 | TGGCTGGAGGGTATGACGAG | TTGCCTTGAAATCCTTGAGGG | 130 | CA | (Daly *et al.*, 2012) |
| Endoglin | - | ATCCTCCTGTCTCAACTGGC | TGGATCTGGGGAGTAGCTCA | 91 | MP | (Zhang *et al.*, 2013) |
| FAP | Fibroblast activation protein | GGAAGTGCGTCATGTGGGTA | GCACCAGTTCTATCTGGGCT | 138 | MS | This study |
| FN1 | Fibronectin 1 | GCTCAGCAAATCGTGCAGC | CCATAGCAGGTACAAACCAGG | 143 | CT | This study |
| FoxJ1 | Forkhead box protein J1 | GGTCTCAGGCACACAAACCT | CCGGGACTCTTTCCAGCAAT | 257 | EL | This study |
| Fsp1 | Fibroblast specific protein | TCCACCCCATCCAAGTCTCT | TTGGGGAGAGCCCAACAATC | 227 | MS | (Lawson *et al.*, 2005) |
| GAPDH | Glyceraldehyde-3-phosphate dehydrogenase | TGTGTCCGTCGTGGATCTGA | CCTGCTTCACCACCTTCTTGA | 77 | HK | (Lin *et al.*, 2010) |
| MMP3 | Matrix metalloproteinase 3 | GGAGCTTCTGCAGGTAAGGG | GTTGCCTCTGCCACCATACT | 227 | TM | This study |
| MUC5AC | Mucin 5AC | GTG CAGGGC TCA GTT CTT TC | TGA CCC AGA TCC TCC ATCTC | 537 | GC | This study |
| Osteop | Osteopontin | AGCAAGAAACTCTTCCAAGCAA | GTGAGATTCGTCAGATTCATCCG | 134 | OB | (Daly *et al.*, 2012) |
| Pkg1 | Protein kinase G | CTGACTTTGGACAAGCTGGACG | GCAGCCTTGATCCTTTGGTTG | 110 | HK | (Veazey *et al.*, 2011) |
| Sca-1/Ly6 | Stem cell marker 1 | AGGTAATGTGGGTGGCTGAAA | GTCCCCCTGTTGAGTCCAAT | 130 | MP | (Peister *et al.*, 2004) |
| Scgb3a2 | Secretoglobin family 3A member 2 | GGCGGTTCCAGTCAGCTAAA | AAGAGCATGCAGGGCAATCT | 301 | CS | This study |
| Sdha | Succinate dehydrogenase complex, subunit A, flavoprotein (Fp) | GCTCCTGCCTCTGTGGTTGA | AGCAACACCGATGAGCCTG | 134 | HK | (Veazey *et al.*, 2011) |
| SPA | Surfactant protein A | TGAGGGCTGACTGGGTAGAA | CCACCAGCCAATCTGTAGCA | 515 | AII | This study |
| SPB | Surfactant protein B | CTGCTTCCTACCCTCTGCTG | CTTGGCACAGGTCATTAGCTC | 175 | AII | This study |
| SPC | Surfactant protein C | CGACCCTGTGAAGCAAAGAAC | TTCCGAGTCCGATTCTTCCG | 306 | AII | This study |
| SPD | Surfactant protein D | CTCTAGTTGTGGCAGCCCTT | GTTAGTGGGGCCTTTCTCCC | 384 | Lung | This study |
| Spdef | Sam pointed domain-containing Ets transcription factor | GCTGACCTCGAAATGGCTGT | TGACTAAGAGGCCACCACCT | 130 | GC | This study, (Rock *et al.*, 2011b) |
| Stan | Stanniocalcin | ATGTGTCTCTGTGACGGCTG | AGGCTTATAGGGAACGGGGT | 501 | TM | This study |
| Tbp | TATA box binding protein | GAAGAACAATCCAGACTAGCAGCA | CCTTATAGGGAACTTCACATCACAG | 127 | HK | (Veazey *et al.*, 2011) |
| TGF-B | Transforming growth factor- beta | CAACAATTCCTGGCGTTACCTTGG | GAAAGCCCTGTATTCCGTCTCCTT | 128 | CK | (Huang *et al.*, 2008) |
| Trp63 | Transformation related protein 63 | GCCTCCCATGTCAGTTTCTCC | GATTACCTGCCCATGCTCACT | 186 | BS | This study, (Rock *et al.*, 2011b) |
| TTF1/NKX2-1 | Thyroid transcription factor 1/NK2 homeobox 1 | GAAGCCATGCAGAGGTTAG | GCCAAATCTAATAGCCACACC | 261 | EL | (Daly *et al.*, 2012) |
| Vim | Vimentin | GTGCGCCAGCAGTATGAAAG | GCATCGTTGTTCCGGTTGG | 110 | MS | (Linke *et al.*, 2009) |
| Ymhaz | Tyrosine 3-monooxygenase/tryptophan 5-monooxygenase activation protein, zeta polypeptide | TTGATCCCCAATGCTTCGC | CAGCAACCTCGGCCAAGTAA | 88 | HK | (Veazey *et al.*, 2011) |
| ZO1 | Zonula occludens 1 | GGTTGTTCTTCGAGAAGCTG | CACCTTGCTTAGAGTCAGG | 302 | EC | (Daly *et al.*, 2012) |

We acknowledge that these markers are expressed in other tissues as well, but for the defined purpose of this study, we will focus on their role listed in this table. AII = Alveolar type II, CS = Clara cell, AC = Adipocyte, EC = Epithelial cell, GC = Goblet cell, CA = Cartilage, OB = Osteoblast, HK = Housekeeping gene, EL = Early lung differentiation marker, CK = Cytokine, MS = Mesenchymal cell, MP = Multipotency marker, TM = MSC tumor marker
